# Early dexmedetomidine and postoperative RASS-defined examinability after microsurgical clipping for aneurysmal subarachnoid hemorrhage: a time-resolved 24-h landmark cohort study

**DOI:** 10.3389/fneur.2026.1908727

**Published:** 2026-09-04

**Authors:** Guilan Ding, Xue Li, Jinliang Luo, Lin Tian, Qing Lan, Wenlai Zhou, Xiangde Zheng

**Affiliations:** 1 Department of Critical Care Medicine, Dazhou Central Hospital, Dazhou, Sichuan, China; 2 Department of Clinical Research Center, Dazhou Central Hospital, Dazhou, Sichuan, China; 3 Department of Geriatrics, Dazhou First People's Hospital, Dazhou, Sichuan, China

**Keywords:** aneurysmal subarachnoid hemorrhage, bradycardia, dexmedetomidine, neurological examinability, nimodipine, ordered-target attainment, Richmond agitation-sedation scale, sedation-state trajectory

## Abstract

**Background:**

After microsurgical clipping for aneurysmal subarachnoid hemorrhage (aSAH), sedation should preserve neurological examinability without compromising hemodynamics or nimodipine delivery. We examined whether early postoperative dexmedetomidine was associated with a greater post-landmark RASS-defined examinability.

**Methods:**

We retrospectively reviewed adults with aSAH treated with clipping at one center from October 2021 to October 2025. Early dexmedetomidine was initiation within 24 h after clipping and continuation for at least 2 h. Exposure was classified during 0–24 h. The primary endpoint was the time-weighted proportion of observed 24–72 h time in RASS-defined examinability defined without reference to the contemporaneous ordered target (Richmond Agitation-Sedation Scale [RASS] −2 to 0). Ordered-target attainment was the key secondary endpoint. Hour-level states were modeled in consecutive 6-h bins using generalized estimating equations (GEE); target-conditional and shared examinable-target-band analyses tested whether easier prescription targets explained the association. The endpoint hierarchy was finalized after data analysis during peer review; the study was not preregistered, no multiplicity adjustment was applied, and all estimates are exploratory.

**Results:**

Among 123 eligible patients, 120 were alive and classifiable at 24 h; 41 received early dexmedetomidine and 79 did not. Examinable time during 24–72 h was 68% versus 55% (Hodges-Lehmann location shift, 12 percentage points; 95% CI, 5–19), and the GEE-adjusted marginal difference was 11.8 points (95% CI, 4.1–19.5). Early users had less post-landmark deep-sedation time; post-landmark co-sedative exposure, dose, and duration differences were directionally lower but imprecise. Ordered-target attainment was directionally concordant; the primary association remained after adding ordered-target stratum and in the shared-target restriction. Window-specific differences were 15.2 points (95% CI, 6.0–24.4) and 6.3 points (95% CI, −2.4 to 15.0); the exposure-by-window interaction was imprecise (difference-in-differences, 8.9 points; 95% CI, −3.6 to 21.4; *p* = 0.164). Bradycardia was more frequent (22.0% vs. 7.6%; risk difference, 14.4 points; 95% CI, 1.6–29.7), whereas other safety estimates were imprecise.

**Conclusion:**

The principal finding was greater post-landmark RASS-defined examinability; related care-process and co-sedative measures were not independent validation. Its descriptive concentration during 24–48 h was not confirmed by the formal interaction test and remains hypothesis-generating, with bradycardia as the principal trade-off. The findings remain vulnerable to confounding by indication and do not establish a drug-specific causal effect or downstream clinical benefit.

## Introduction

Aneurysmal subarachnoid hemorrhage (aSAH) remains a high-risk neurocritical-care disease despite advances in early aneurysm occlusion, nimodipine therapy, cerebral vasospasm surveillance, and complication management (1–4). Early outcomes are driven by the initial hemorrhage and early brain injury, while later deterioration may reflect delayed cerebral ischemia (DCI), hydrocephalus, infection, systemic organ dysfunction, and the quality of coordinated ICU care (1–4). In patients treated with clipping, the immediate postoperative ICU period is a narrow interval in which sedation, blood pressure, respiratory support, and serial neurological assessment interact directly.

Sedation in aSAH is not a static exposure but a sequence of clinically meaningful patient states and care-process decisions. Deep sedation may be appropriate for intracranial-pressure control or ventilator synchrony, yet prolonged non-examinability can obscure neurological deterioration; agitation can be equally hazardous. The clinically relevant patient-state question is whether the patient is actually within a RASS range compatible with examination. A separate care-process question is whether the bedside team achieves the target it ordered. These endpoints are complementary rather than interchangeable (5).

Evidence for postoperative sedation strategies that preserve examinability is sparse. A recent scoping review identified only 11 eligible studies, with small samples and heterogeneous physiological or clinical endpoints, and judged the evidence for analgosedative choice in aSAH critically inadequate (6). Survey data likewise show heterogeneous sedation practice and no specific protocol recommendations, while interruption of prolonged sedation can itself provoke frequent adverse events (7, 8). Prior dexmedetomidine studies have mainly addressed intraoperative physiology, biomarkers, neurological outcomes, or mortality (9–17), rather than the post-clipping trajectory of RASS states and the sedatives used to produce them.

Accordingly, this study used a hierarchical endpoint framework. Exposure was classified during 0–24 h. The primary endpoint was the proportion of observed time in RASS-defined examinability, defined without reference to the contemporaneous ordered target, (RASS −2 to 0) during 24–72 h; ordered-target attainment over the same post-landmark interval was retained as the key secondary care-process endpoint and analyzed conditional on target stratum. Deep-sedation burden, agitation, and displacement of propofol or midazolam characterized how the state was achieved, while bradycardia, hypotension, vasopressor escalation, and nimodipine delivery defined the principal trade-offs. The estimand is strategy-associated and observational, not a drug-specific causal effect.

### What this study adds

This study combines two related layers of evidence that administrative datasets cannot recover: a patient-state endpoint defined without reference to the prescribed target, and a target-relative process endpoint that measures execution of the sedation plan. Separation of exposure and outcome windows, 6-h RASS trajectories, target-conditional and shared examinable-target-band analyses, sedative displacement, proximal nimodipine delivery, and time-resolved safety characterization provide a clinically interpretable strategy profile rather than a generic drug-use description.

## Materials and methods

### Study design and setting

We performed a single-center retrospective cohort study at Dazhou Central Hospital and followed the Strengthening the Reporting of Observational Studies in Epidemiology (STROBE) framework (18). The source population included adults with aSAH admitted to the ICU between October 2021 and October 2025 and treated with early microsurgical clipping. Postoperative ICU admission after aneurysm clipping was time zero. A completed STROBE checklist, a variable-level missingness matrix, and detailed cohort-exclusion counts are provided in the Supplementary material.

Because early dexmedetomidine status could only be assigned after the first postoperative day had been observed, the analytic cohort was restricted to patients alive and classifiable at 24 h. Exposure was defined from 0 to 24 h, whereas the primary sedation outcome was assessed from 24 to 72 h. Values from 0 to 24 h were retained only as descriptive exposure-window information. This separation protects the principal process analysis from the direct overlap between treatment classification and outcome ascertainment, although it does not eliminate confounding by indication.

### Participants

Patients were eligible if they were 18 years or older, had aSAH confirmed by computed tomography, computed tomographic angiography, digital subtraction angiography, or operative findings, were admitted within 72 h after ictus, underwent microsurgical clipping within 48 h after admission, and had ascertainable 60-day vital status.

**Figure 1:**
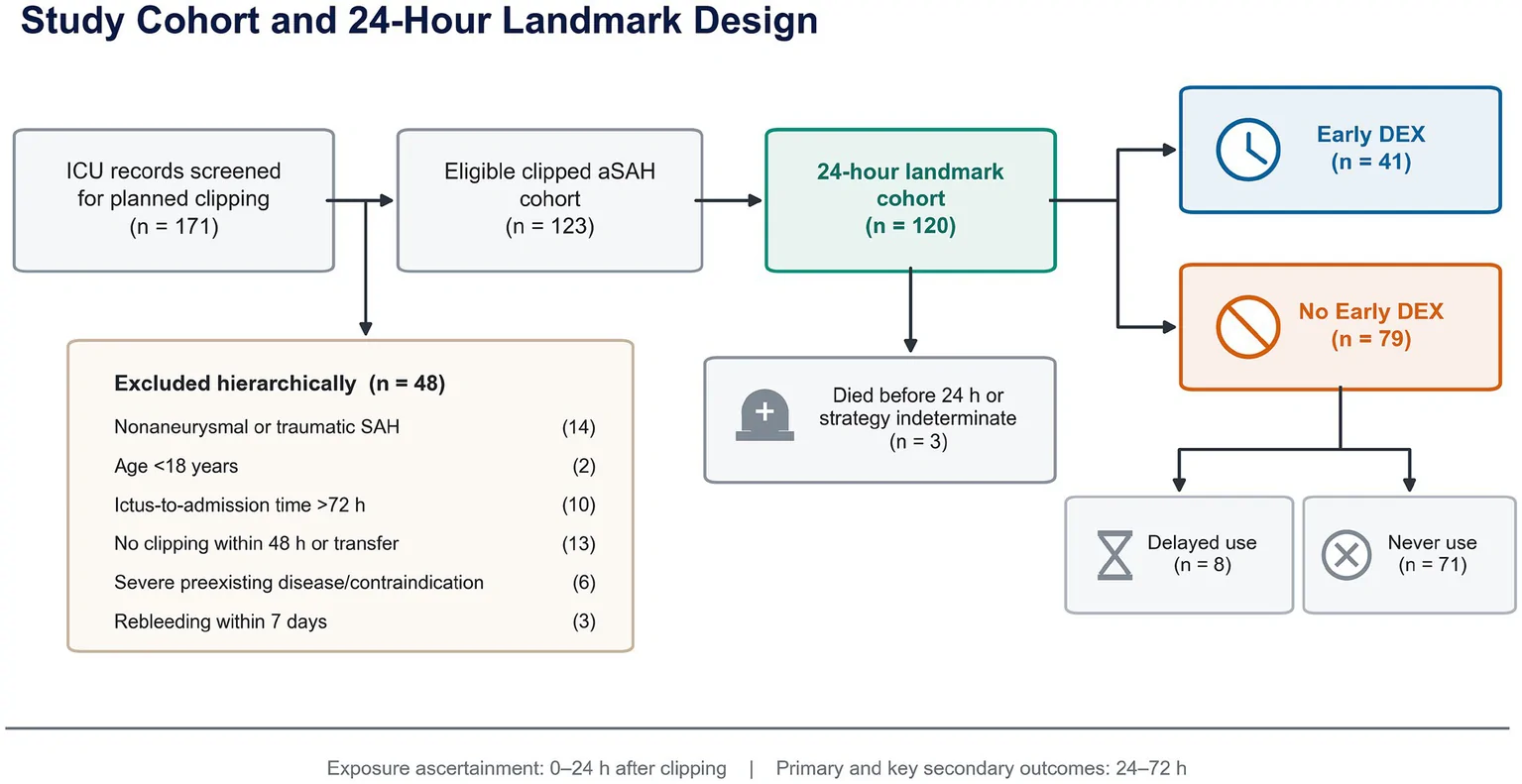
Cohort construction and exposure classification. The flow diagram shows the 171 screened ICU records, mutually exclusive exclusion categories, the 123 eligible clipped-aSAH patients, the three patients excluded before the 24-h landmark, and the 120-patient landmark cohort. The no-early group is subdivided into eight delayed dexmedetomidine users and 71 never users.

We excluded patients with non-aneurysmal or traumatic subarachnoid hemorrhage, age younger than 18 years, delayed admission beyond 72 h, no clipping within 48 h or transfer elsewhere for definitive treatment, severe pre-existing neurological disability, terminal organ failure, active malignancy, severe acute hepatic or renal injury, severe pneumonia or intracranial infection before admission, recent corticosteroid or immunosuppressive exposure, chronic alcohol or sedative-hypnotic misuse, rebleeding within 7 days, or unavailable exposure or outcome information. Exclusion categories were applied hierarchically and are shown as mutually exclusive counts in Figure 1. Day-60 vital status and modified Rankin Scale (mRS) were ascertained from hospital records and structured follow-up contacts; all 120 landmark patients had complete day-60 ascertainment.

### Perioperative care and exposure definition

Patients received standard postoperative neurocritical care, including blood-pressure management, enteral nimodipine when tolerated, analgesia and sedation, temperature control, infection prevention, nutrition, and serial neurological assessment. Surveillance for vasospasm and ischemia used transcranial Doppler, computed tomography, vascular imaging, perfusion imaging, electroencephalography, or other monitoring according to clinical need. During the study period, RASS was scheduled at least every 4 h and after clinically important sedative changes; the usual examinable target was RASS −2 to 0 unless intracranial pressure, ventilator synchrony, or another documented indication required deeper sedation. Enteral nimodipine was generally prescribed as 60 mg every 4 h, with 30 mg every 2 h considered before interruption when hypotension limited delivery.

Dexmedetomidine was prepared at 4 micrograms/mL and usually administered without a loading dose. Infusion commonly started at 0.2–0.4 micrograms/kg/h and was titrated between 0.2 and 0.8 micrograms/kg/h according to the ordered RASS target, heart rate, blood pressure, ventilation, and the need for neurological examination.

Early dexmedetomidine was defined as initiation within 24 h after clipping and continuation for at least 2 h. The principal comparator included patients who never received dexmedetomidine and patients who started after 24 h; a restricted analysis compared early users with never users. Exposure-definition sensitivity analyses used initiation cutoffs of 12 h and 36 h and a minimum continuation threshold of 4 h. Admission period was included in adjusted analyses to account for possible practice changes from 2021 to 2025. Operational definitions for exposure, landmark timing, endpoints, and safety outcomes are summarized in Supplementary Table S1.

### Endpoint hierarchy and estimands

Time within the contemporaneous ordered RASS target may partially embed treatment intention in the outcome. The primary endpoint is order-independent only by definition; it is not empirically independent of the prescribed target. Initiating dexmedetomidine and ordering a lighter target may arise from the same bedside decision. The primary endpoint was therefore defined as the absolute RASS-defined patient state during the non-overlapping 24–72 h interval. This endpoint hierarchy separates the patient-state outcome from prescription preference and reduces order-dependent circularity; it does not eliminate confounding by indication in treatment allocation. Ordered-target attainment was retained as the key secondary care-process endpoint.

The primary estimand was the between-group difference in the patient-level time-weighted proportion of classifiable post-landmark hours in RASS-defined examinability (RASS −2 to 0) among patients alive and classifiable at 24 h. The key secondary estimand was the difference in time-weighted ordered-target attainment during 24–72 h, interpreted as care-process performance and evaluated conditional on target stratum. Supporting state estimands were deep-sedation burden (RASS ≤ −4), agitation burden (RASS ≥ +1), and examinable time without concurrent propofol or midazolam. Time-specific marginal differences described whether the association was concentrated in the first or second post-landmark day. Safety estimands were descriptive risk differences or median differences; overlap-weighted mortality remained exploratory.

### Sedation-state trajectory and hemodynamic/nimodipine outcomes

The primary endpoint was the percentage of observed post-landmark time (24–72 h) in RASS-defined examinability, defined as RASS −2 to 0 irrespective of the contemporaneous order. Each RASS entry was carried forward from its charted time until the next assessment, capped at 6 h. Intervals beyond 6 h, periods outside ICU observation, and intervals without a classifiable RASS value were excluded from the denominator. The same hour-level reconstruction classified deep sedation (RASS ≤ −4), moderate non-examinable sedation (RASS −3), examinability (RASS −2 to 0), and agitation (RASS ≥ +1). State occupancy was summarized overall and in consecutive 6-h bins from 24 to 72 h. Variable-level completeness and RASS observation coverage are reported in Supplementary Table S2. Boundary sensitivity analyses used RASS −2 to +1 and RASS −3 to +1. A risk-set-aligned index-state model used each early user’s last RASS recorded within 2 h before dexmedetomidine initiation. Each comparator was assigned one pseudo-index hour by deterministic quantile matching to the empirical distribution of early-user initiation times and contributed the last RASS within the preceding 2 h while still untreated; the resulting index-state indicator was included as a covariate. The 0–24 h patient-level proportion remained descriptive because it could include post-initiation time. Numbers contributing to each 6-h bin were tabulated; sensitivity analyses carried death forward as non-examinable, restricted the analysis to patients with at least 24 h of analyzable time, and used an alive-and-examinable composite. In the composite, death or withdrawal was coded non-examinable through 72 h and ICU discharge alive was carried forward as examinable through 72 h; this was a directional sensitivity analysis, not a patient-centered outcome. Documented Glasgow Coma Scale, pupillary, and motor examinations were counted as a RASS-independent documentation-density check, not as a blinded or direct measure of examination quality.

**Figure 2:**
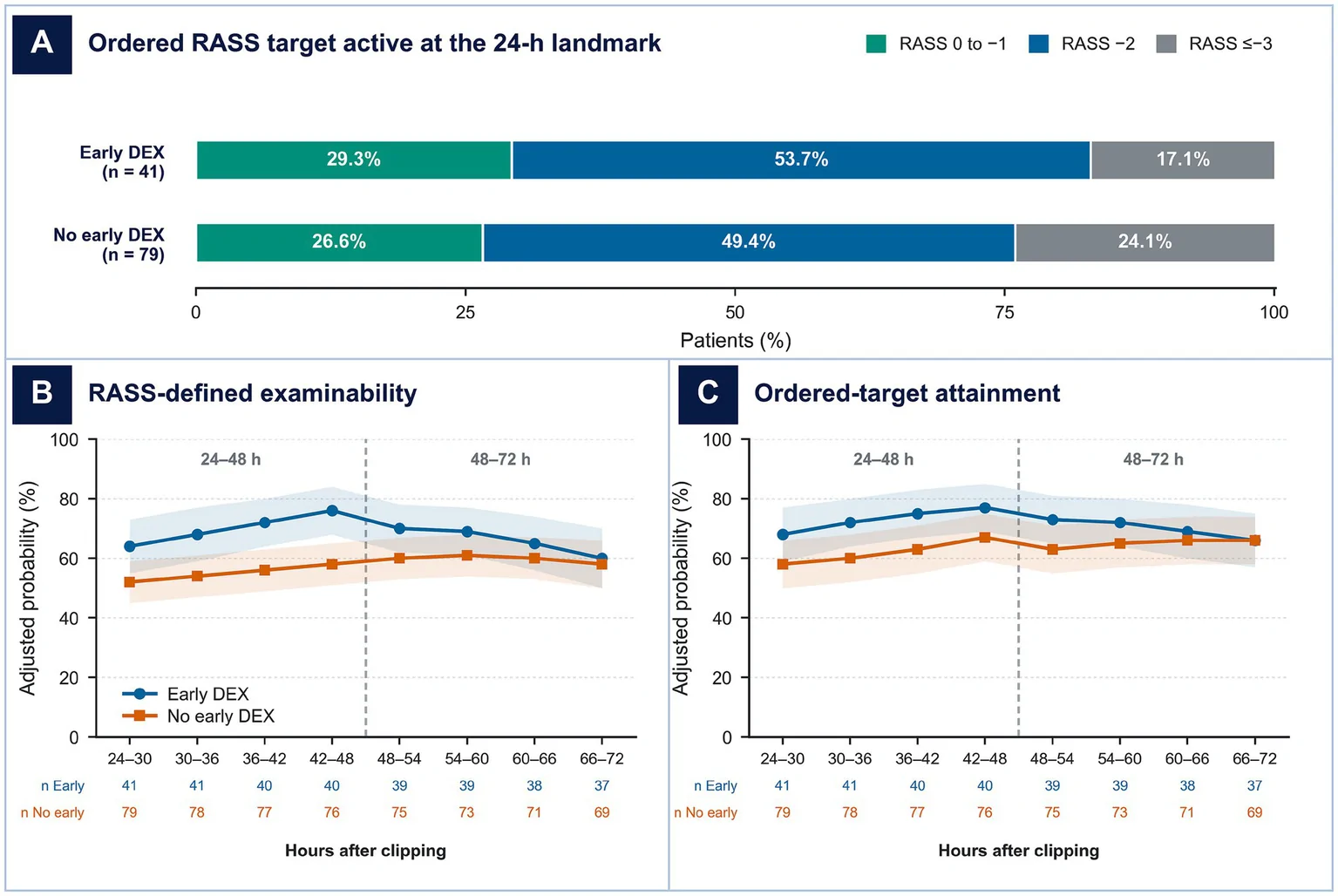
Hierarchical sedation endpoints and time-resolved trajectories. **(A)** Distribution of the ordered RASS target active at the 24-h landmark. **(B)** Adjusted marginal probability of the primary endpoint, RASS-defined examinability (RASS −2 to 0), in consecutive 6-h bins from 24 to 72 h. **(C)** Adjusted marginal probability of ordered-target attainment over the same bins. In panels B and C, the horizontal axis reports hours after clipping, shaded bands show 95% confidence intervals, and the numbers below each bin report patients contributing observations. Window-specific trajectories are descriptive; the exposure-by-window interaction was imprecise (*p* = 0.164).

The key secondary endpoint was the time-weighted proportion of observed 24–72 h time within the contemporaneous ordered RASS target. Target category, target changes, RASS assessment density, and analyzable-time coverage were reported to distinguish care-process performance from differences in prescription difficulty or observation intensity. A target-conditional GEE treated target category as a contextual covariate, and a restriction analysis included patients whose ordered target at the 24-h landmark was RASS −2 to 0. Because target selection may itself be part of the treatment strategy, these analyses were considered diagnostic rather than causal adjustment. Additional secondary outcomes included examinable time free of active propofol or midazolam and the exposure, dose, and duration of propofol, midazolam, and continuous opioids. Co-sedative exposure, cumulative dose, and infusion duration were restricted to 24–72 h; values from 0 to 24 h were not used as outcome evidence. Ordered-target distributions and monitoring diagnostics are reported in Table 1, with the landmark target distribution shown in Figure 2A.

**Table 1:** Endpoint hierarchy, time-resolved RASS trajectories, ordered-target performance, and core robustness analyses.

Variable	Early dexmedetomidine (*n* = 41)	No early dexmedetomidine (*n* = 79)	Effect estimate (95% CI)
Dexmedetomidine exposure			
DEX recipients in group	41 (100)	8 (10.1), delayed users	--
DEX start time after surgery, h	6.5 (2.8–12.0)	48 (31–66)	--
DEX duration, h	38 (18–72)	20 (10–44)	--
Median infusion rate, micrograms/kg/h	0.42 (0.30–0.58)	0.36 (0.25–0.52)	--
Primary patient-state endpoint and temporal profile			
RASS-defined examinability, 24–72 h (RASS −2 to 0)	68 (55–80)%	55 (39–72)%	Hodges-Lehmann location shift +12 points (95% CI, +5 to +19)
GEE-adjusted marginal probability, 24–72 h	69%	57%	Average marginal difference +11.8 points (95% CI, +4.1 to +19.5)
Risk-set-aligned pre-initiation-state GEE	68%	58%	Average marginal difference +10.1 points (95% CI, +2.4 to +17.8)
Target-conditional GEE for examinability	67%	59%	Average marginal difference +7.8 points (95% CI, +1.4 to +14.2)
Shared examinable-target-band restriction	70% (*n* = 34)	60% (*n* = 60)	Average marginal difference +9.6 points (95% CI, +2.0 to +17.2)
Temporal attenuation in adjusted examinability	--	--	24–48 h: +15.2 points (95% CI, +6.0 to +24.4); 48–72 h: +6.3 points (95% CI, −2.4 to +15.0); interaction contrast +8.9 points (95% CI, −3.6 to +21.4), *p* = 0.164
Key secondary care-process endpoint			
Ordered-target attainment, 24–72 h	72 (59–84)%	64 (48–78)%	Median difference +8 points (95% CI, +2 to +14)
Target-conditional GEE for ordered-target attainment	71%	64%	Average marginal difference +7.1 points (95% CI, +1.2 to +13.0)
Shared examinable-target-band restriction: ordered-target attainment	74%	66%	Difference +8.2 points (95% CI, +1.0 to +15.4)
Ordered-target attainment by post-landmark window	24–48 h: 73 (58–86)%; 48–72 h: 70 (54–82)%	24–48 h: 62 (45–78)%; 48–72 h: 65 (46–80)%	24–48 h: +11 points (95% CI, +4 to +18); 48–72 h: +5 points (95% CI, −2 to +12)
Supporting state occupancy and sedative displacement			
Propofol cumulative dose and infusion duration, 24–72 h	8.6 (0–24.0) mg/kg; 10 (0–30) h	15.4 (0–35.6) mg/kg; 18 (0–46) h	Dose difference −5.8 mg/kg (95% CI, −13.9 to +1.0); duration difference −6 h (95% CI, −16 to +2)
Midazolam cumulative dose and infusion duration, 24–72 h	0.0 (0–0.8) mg/kg; 0 (0–3) h	0.0 (0–2.1) mg/kg; 0 (0–8) h	Dose difference −0.2 mg/kg (95% CI, −0.8 to +0.1); duration difference −2 h (95% CI, −8 to +1)
Continuous-opioid cumulative dose and infusion duration, 24–72 h	0.12 (0.00–0.28) morphine-equivalent mg/kg; 12 (0–32) h	0.15 (0.00–0.36) morphine-equivalent mg/kg; 18 (0–40) h	Dose difference −0.03 mg/kg (95% CI, −0.10 to +0.04); duration difference −4 h (95% CI, −12 to +4)
Deep-sedation time, 24–72 h (RASS ≤ −4)	10 (3–22)%	21 (8–34)%	Median difference −11 points (95% CI, −18 to −4)
Agitation time, 24–72 h (RASS ≥ +1)	6 (0–12)%	10 (2–19)%	Median difference −4 points (95% CI, −9 to +1)
Examinable time without active propofol or midazolam, 24–72 h	52 (34–71)%	34 (18–54)%	Median difference +17 points (95% CI, +7 to +28)
Core indication, observation, and robustness diagnostics			
Propofol exposure, 24–72 h	18 (43.9)	47 (59.5)	RD −15.6 percentage points (95% CI, −32.9 to +3.1)
Midazolam exposure, 24–72 h	8 (19.5)	25 (31.6)	RD −12.1 percentage points (95% CI, −26.4 to +5.0)
Continuous opioid infusion, 24–72 h	15 (36.6)	38 (48.1)	RD −11.5 percentage points (95% CI, −28.4 to +7.1)
Documented neurological examinations, 24–72 h	10 (8–12)	10 (8–12)	Hodges-Lehmann location shift 0 examinations (95% CI, −1 to +1)
Landmark ordered-target distribution	0 to −1: 12 (29.3); −2: 22 (53.7); ≤ −3: 7 (17.1)	0 to −1: 21 (26.6); −2: 39 (49.4); ≤ −3: 19 (24.1)	No category showed a clearly important imbalance
Analyzable-time coverage, 24–72 h	94% (88–100)	92% (84–100)	Median difference +2 points (95% CI, −1 to +5)
Early versus never use: full process and co-sedative domain	Early users (*n* = 41)	Never users (*n* = 71)	Examinability +11.4 points (95% CI, +3.5 to +19.3); ordered target +9 (+2 to +16); deep sedation −12 (−19 to −5); propofol/midazolam-free examinability +18 (+7 to +29); propofol/midazolam/opioid exposure RDs − 18.1 (−35.5 to +0.6)/−14.3 (−29.2 to +3.0)/−12.7 (−30.1 to +6.2) points. Full results: Supplementary Table S4.
Alternative initiation/continuation definitions	--	--	Adjusted differences ranged from +9.8 to +12.7 percentage points
Alternative carry-forward definitions	--	--	Adjusted differences ranged from +10.2 to +12.4 points
Calendar-period interaction	--	--	No material interaction; exposure-by-period *p* = 0.64

Continuous variables are median (interquartile range), and categorical variables are *n* (%). Effect estimates are early minus no early dexmedetomidine with 95% CIs unless otherwise stated. The primary endpoint is RASS-defined examinability during 24–72 h, defined without reference to the ordered target; ordered-target attainment is the key secondary care-process endpoint. Target-conditional, shared-target, early-versus-never (covering state, target, and co-sedative endpoints), exposure-definition, and carry-forward analyses are diagnostic robustness checks and do not eliminate confounding by indication. DEX, dexmedetomidine; GEE, generalized estimating equation; RASS, Richmond Agitation-Sedation Scale.

Hemodynamic safety outcomes included bradycardia, clinically relevant hypotension, vasopressor escalation, norepinephrine-equivalent exposure, and dexmedetomidine dose reduction or interruption. Bradycardia was heart rate below 50 beats/min for at least 5 consecutive minutes or prompting dose reduction, interruption, or treatment, ascertained from continuous bedside monitoring and charted vital-sign records. Clinically relevant hypotension was systolic blood pressure below 90 mmHg or mean arterial pressure below 65 mmHg for at least 15 min and prompting fluid administration or vasopressor escalation; nimodipine reduction alone did not define hypotension. Nimodipine reduction for hypotension and interruption longer than 24 h were analyzed separately and were not combined with hypotension into a composite. Nimodipine delivery was summarized only over 24–72 h as an exposure-proximal descriptor. The planned-dose denominator comprised doses scheduled by the active order while patients were alive and hospitalized during 24–72 h; doses remained eligible after ICU transfer and tube-to-oral transition, whereas doses scheduled after death or hospital discharge did not. A 30-mg every-2-h regimen was converted to 60-mg every-4-h-equivalent planned opportunities. Because the denominator can end after a terminal event, this measure was not interpreted as a survivor-comparable outcome. Among early users, time to first bradycardia was displayed without post-baseline rate stratification; a start-stop time-varying infusion-rate model replaced the whole-infusion median-rate analysis.

### Exploratory downstream clinical outcomes

Exploratory downstream outcomes included all-cause mortality by 60 days after surgery, in-hospital mortality, delayed cerebral ischemia (DCI), symptomatic cerebral vasospasm, new ischemic infarction, favorable day-60 functional status defined as modified Rankin Scale (mRS) 0–3, mechanical ventilation duration, ICU length of stay, and hospital-acquired infection. These outcomes were retained to show whether the process phenotype was accompanied by an obvious downstream signal, but they were not used to define the central claim. Hospital-acquired infection was retained as an exploratory falsification-style outcome because no direct short-term protective effect was expected; it was not regarded as a strict negative control because sedation could indirectly affect ventilation duration or infection risk. The full day-60 mRS distribution is shown in Supplementary Figure S1.

DCI was classified using consensus principles: a new sustained focal deficit, reduction in Glasgow Coma Scale score, or new ischemia or infarction on imaging during the typical post-aSAH risk window, after excluding rebleeding, hydrocephalus, seizure, infection, metabolic disturbance, sedative effect, and procedure-related infarction (19). DCI was adjudicated from routine clinical documentation and imaging rather than by a blinded prospective committee. Diagnostic source was classified as imaging-supported or clinical-only, and ascertainment bias related to examinability was considered explicitly.

### Statistical analysis

Continuous variables are reported as mean with standard deviation or median with interquartile range. Categorical variables are reported as number and percentage. Baseline imbalance was described with standardized mean differences (SMDs), using 0.10 as a descriptive threshold rather than a significance test. Categorical contrasts are reported primarily as risk differences with Newcombe 95% CIs and secondarily as risk ratios where clinically useful. Median differences and their 95% CIs were estimated with the Hodges-Lehmann approach or 10,000 stratified bootstrap resamples, as appropriate. No multiplicity correction was applied; all process and safety CIs are descriptive. The endpoint hierarchy was finalized after data analysis during peer review, no protocol was preregistered, and all analyses are exploratory and hypothesis-generating.

**Figure 3:**
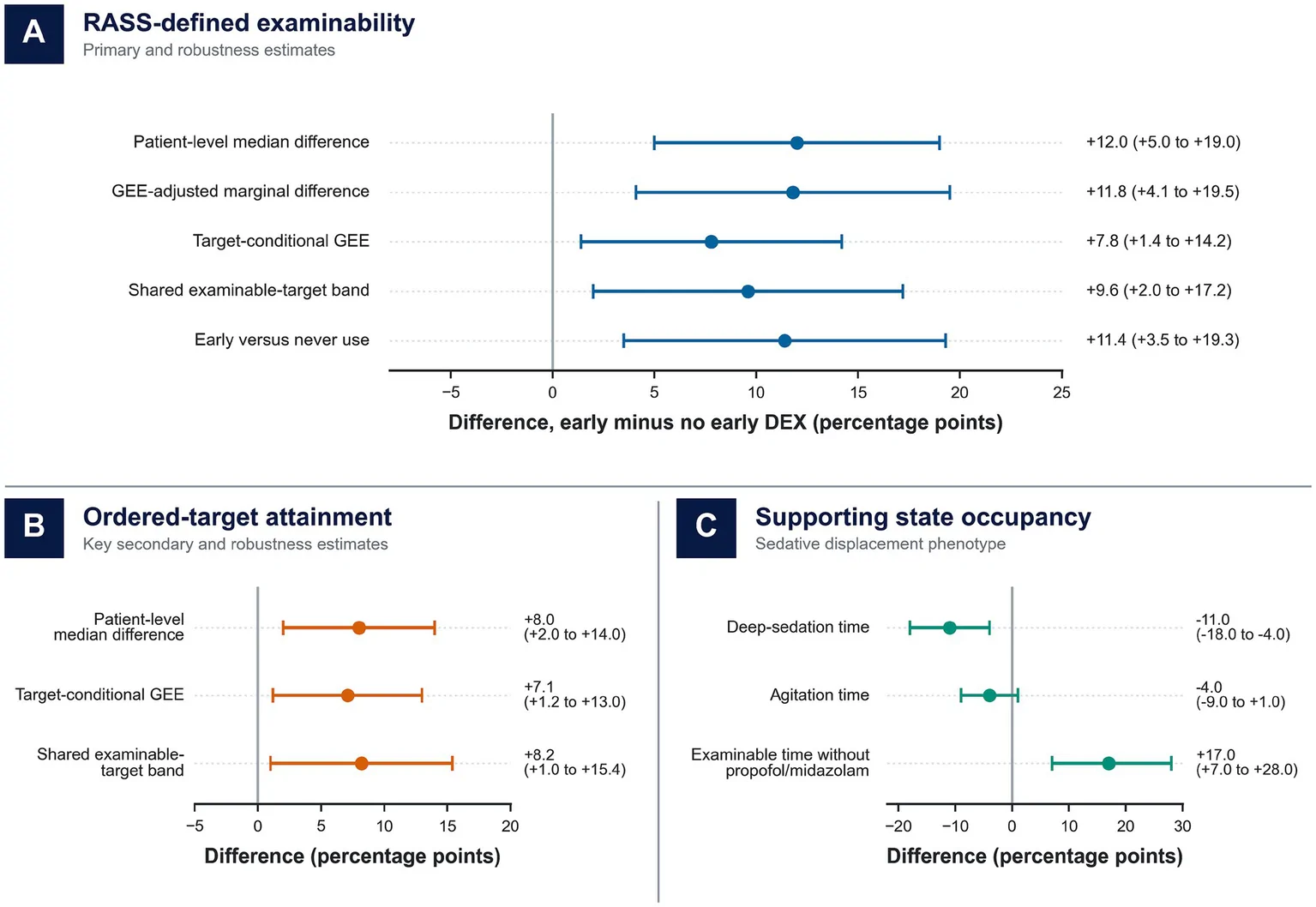
Patient-state, care-process, and sedative-displacement estimates. **(A)** Primary examinability and robustness estimates. **(B)** Ordered-target attainment estimates from the unadjusted, target-conditional, and shared examinable-target-band analyses. **(C)** Post-landmark deep-sedation, agitation, and propofol/midazolam-free examinability differences. Each panel reports early minus no early dexmedetomidine in percentage points with 95% confidence intervals; incompatible units are not combined.

The primary patient-level contrast was the between-group median difference in post-landmark examinable time. The main population-averaged GEE modeled binary hour-level examinability with robust standard errors, an autoregressive working correlation, consecutive 6-h bins, early dexmedetomidine, an exposure-by-time interaction, admission period, APACHE II score, WFNS grade IV-V, mechanical ventilation at ICU admission, and norepinephrine use at ICU admission. Marginal standardization yielded the adjusted average probability difference over 24–72 h and window-specific differences for 24–48 h and 48–72 h. The formal exposure-by-window interaction was reported as the difference between these two marginal contrasts with its 95% CI and *p* value. The target-conditional sensitivity model explicitly added the ordered-target stratum active at the 24-h landmark; because this target could reflect the same clinical strategy that generated exposure, the model tested robustness to prescription preference rather than estimating a controlled direct effect. The key secondary ordered-target endpoint was analyzed with a parallel GEE that included ordered-target stratum and the same baseline covariates. A shared examinable-target-band restriction included patients whose 24-h landmark ordered target fell within RASS −2 to 0. Additional sensitivity analyses compared early users with never users across the primary examinability endpoint, ordered-target attainment, deep-sedation and agitation time, examinable time without active propofol or midazolam, and propofol, midazolam, and continuous-opioid exposure; exposure and carry-forward definitions were also varied. The principal and diagnostic process estimates are summarized in Table 1 and Figure 3. A process-endpoint sensitivity analysis additionally applied the mortality-model overlap weights to the longitudinal GEE, targeting the ATO population.

The mortality propensity score was estimated with logistic regression using prespecified baseline or pre-exposure variables: admission period, age, sex, APACHE II score, WFNS grade IV-V, modified Fisher grade 3–4, acute hydrocephalus, mechanical ventilation at ICU admission, norepinephrine at ICU admission, intraoperative rupture, surgery duration, and ictus-to-admission time. Age, APACHE II score, surgery duration, and ictus-to-admission time were modeled with restricted cubic splines with three knots. No post-exposure RASS measure or co-sedative was included. There were no missing propensity-score covariates. Exposed patients received weights of 1 minus the propensity score and unexposed patients received weights equal to the propensity score. Weighted logistic regression with a robust sandwich variance estimator produced the ATO mortality odds ratio and 95% CI (20, 21). No interaction terms were included; weights were not stabilized, trimmed, or truncated, and no common-support exclusions were applied. The unweighted longitudinal GEE targets the full landmark cohort, whereas the overlap-weighted mortality and process sensitivity models target the ATO population; estimates from these populations are not interpreted as directly comparable treatment effects. Exact mean balance to numerical precision was verified for all propensity-score covariates. Hypertension, diabetes, aneurysm location, aneurysm diameter, white blood cell count, and prealbumin were not included in the score and were retained as descriptive variables. Prealbumin was available for 113/120 patients and was analyzed by available case only; no missing value entered the propensity score.

Overlap and positivity were evaluated with group-specific propensity-score density plots, the range of estimated scores, effective sample size, maximum weight, and pre−/post-weighting SMDs. Numerical propensity-score, weighting, and effective-sample-size diagnostics are reported in Supplementary Table S3, and covariate balance is displayed in Supplementary Figure S2. Weighted estimates were interpreted only as a measured-confounding sensitivity check, not as a substitute for randomization.

No *a priori* sample-size calculation was possible because all eligible cases in the study period were included. With 41 early users and 79 comparators, the study had approximately 80% power at a two-sided alpha of 0.05 to detect a standardized continuous difference of 0.54; smaller process differences, uncommon adverse events, and modest clinical-outcome effects could remain undetected. Repeated hour-level observations increased temporal resolution but did not increase the number of independent patients, so all longitudinal inference remained patient-clustered. Sensitivity analyses included early versus never use, alternative exposure and carry-forward definitions, calendar-period consistency, the falsification-style infection outcome, and E-values for mortality. Mortality timing-window and restriction checks are displayed in Supplementary Figure S3; additional weighting, DCI, E-value, falsification-style outcome, and influence diagnostics are reported in Supplementary Table S3. The full early-versus-never process and co-sedative restriction results are reported in Supplementary Table S4. An exploratory process-endpoint E-value was calculated from the standardized marginal risk ratio rather than the primary probability-difference estimand (22); it was treated as a secondary sensitivity descriptor, not as proof that indication confounding had been removed. The exploratory bradycardia model used time since dexmedetomidine initiation as the time scale, represented infusion rate as a start-stop covariate updated whenever the rate changed, counted only the first bradycardia event, and censored at infusion discontinuation. With nine events, it was unadjusted and interpreted only as a sparse descriptive analysis. Analyses were performed in R version 4.4.2 using the geepack, marginaleffects, WeightIt, cobalt, sandwich, boot, quantreg, and EValue packages.

## Results

### Cohort construction

Among 171 screened ICU records related to subarachnoid hemorrhage and planned clipping, 48 were excluded. The final eligible cohort included 123 patients. Forty-nine patients received dexmedetomidine at some point during perioperative ICU care and 74 did not. Three patients died before the 24-h landmark or had indeterminate early-exposure status. The landmark cohort therefore included 120 patients: 41 with early dexmedetomidine initiation and 79 without early initiation (Figure 1). Of the 79 comparators, 8 started dexmedetomidine after 24 h and 71 never received it. Exposure, primary process outcomes, key safety outcomes, and day-60 vital status were complete; missing RASS observation time is quantified through the analyzable-time coverage measure rather than treated as calendar time at target.

### Baseline characteristics

Baseline characteristics are shown in Table 2. The groups were broadly similar in age, sex, vascular risk factors, ictus-to-admission time, APACHE II score, modified Fisher grade, aneurysm location, hydrocephalus, intraoperative rupture, surgery duration, and early vasopressor use. Early dexmedetomidine patients had slightly more WFNS grade IV-V disease and mechanical ventilation at ICU admission; white blood cell count and prealbumin were also higher. After overlap weighting, all propensity-score covariates were balanced to numerical precision (weighted SMD < 0.001). Weighted SMDs for the untargeted hypertension, diabetes, anterior-circulation location, aneurysm diameter, white blood cell count, and prealbumin descriptors were 0.01, 0.04, 0.02, 0.01, 0.05, and 0.06, respectively. Estimated propensity scores ranged from 0.20 to 0.76 in early users and from 0.08 to 0.73 in comparators; the overlap-weighted effective sample size was 91.4. Pre- and post-weighting balance is shown in Supplementary Figure S2; numerical diagnostics are reported in Supplementary Table S3.

**Table 2:** Baseline characteristics and propensity-score balance in the 24-h landmark cohort.

Variable	Early dexmedetomidine (*n* = 41)	No early dexmedetomidine (*n* = 79)	Unweighted SMD	Weighted SMD
Admission period, 2021–2022	13 (31.7)	33 (41.8)	0.21	0.00
Age, years	58.4 (9.8)	60.3 (10.0)	0.19	0.00
Male sex	20 (48.8)	32 (40.5)	0.17	0.00
Hypertension	20 (48.8)	44 (55.7)	0.14	0.01 (not targeted)
Diabetes mellitus	3 (7.3)	4 (5.1)	0.09	0.04 (not targeted)
Ictus-to-admission time, h	9.1 (6.8)	10.2 (7.0)	0.16	0.00
APACHE II score	22.8 (4.8)	23.4 (5.8)	0.11	0.00
WFNS grade IV-V	26 (63.4)	44 (55.7)	0.16	0.00
Modified Fisher grade 3–4	31 (75.6)	58 (73.4)	0.05	0.00
Anterior circulation aneurysm	33 (80.5)	64 (81.0)	0.01	0.02 (not targeted)
Aneurysm diameter ≥ 7 mm	10 (24.4)	22 (27.8)	0.08	0.01 (not targeted)
Acute hydrocephalus	12 (29.3)	22 (27.8)	0.03	0.00
Intraoperative rupture	5 (12.2)	9 (11.4)	0.02	0.00
Surgery duration, min	230 (69)	237 (86)	0.09	0.00
Mechanical ventilation at ICU admission	31 (75.6)	55 (69.6)	0.13	0.00
Norepinephrine at ICU admission	13 (31.7)	25 (31.6)	0.00	0.00
White blood cell count, x10^9/L	15.7 (5.1)	14.6 (5.3)	0.21	0.05 (not targeted)
Prealbumin, mg/L	247 (53)	230 (47)	0.34	0.06 (not targeted)

Values are mean (SD) or *n* (%). Weighted SMDs are reported after propensity-score overlap weighting for the ATO estimand. Exact balance (<0.001) is expected for covariates in the main-effect logistic propensity-score model; hypertension, diabetes, aneurysm location, aneurysm diameter, white blood cell count, and prealbumin were not included and are labeled as untargeted descriptors. Propensity-score covariates were complete. Prealbumin was available for 113/120 patients and was summarized by available case; no imputation was performed. DEX, dexmedetomidine; SMD, standardized mean difference; WFNS, World Federation of Neurosurgical Societies.

The distribution of ordered RASS targets, RASS monitoring intensity, analyzable-time coverage, and the direction of the adjusted sedation-state contrast did not materially differ between the 2021–2022 and 2023–2025 admission periods; admission period was retained in the longitudinal and propensity-score models.

### Sedation-state trajectories and sedative displacement

In early users, dexmedetomidine started a median of 6.5 h after surgery and continued for a median of 38 h. During 0–24 h, median RASS-defined examinability was 54% versus 51% (median difference, 3 points; 95% CI, −5 to 11); this interval was descriptive because it included post-initiation time in early users. Using the last RASS within 2 h before initiation for early users and one still-untreated comparator RASS observation selected at a deterministic quantile-matched pseudo-index hour, 20/41 (48.8%) early users and 43/79 (54.4%) comparators were within RASS −2 to 0 (risk difference, −5.6 points; 95% CI, −23.5 to 12.8). Eight comparator patients later received dexmedetomidine after the first postoperative day; their median start time was 48 h and median duration was 20 h (Table 1). The ordered target active at the 24-h landmark was RASS 0 to −1 in 12/41 (29.3%) early users and 21/79 (26.6%) comparators, RASS −2 in 22/41 (53.7%) and 39/79 (49.4%), and RASS ≤ −3 in 7/41 (17.1%) and 19/79 (24.1%) (Figure 2A). Target changes occurred in 14.6 and 16.5%, respectively. From 24 to 72 h, the median number of RASS assessments was 12 (IQR, 10–14) and 12 (IQR, 9–14), the median assessment interval was 3.9 and 4.0 h, and analyzable-time coverage was 94 and 92%. Numbers contributing to consecutive 6-h bins were 41/41/40/40/39/39/38/37 in early users and 79/78/77/76/75/73/71/69 in comparators. From 24 to 72 h, there was 1 versus 2 deaths, 2 versus 7 ICU discharges, and 0 versus 1 withdrawal-of-care decision.

During the primary post-landmark interval, early users spent 68% (IQR, 55–80%) of observed time in RASS-defined examinability versus 55% (IQR, 39–72%) among comparators; the Hodges-Lehmann location shift was 12 percentage points (95% CI, 5 to 19). The adjusted average marginal difference was 11.8 percentage points (95% CI, 4.1 to 19.5). Sensitivity estimates were 10.1 points (95% CI, 2.4 to 17.8) after risk-set-aligned pre-initiation-state adjustment, 10.6 points (95% CI, 3.0 to 18.2) using RASS −2 to +1, 8.7 points (95% CI, 1.2 to 16.2) using RASS −3 to +1, and 10.9 points (95% CI, 3.2 to 18.6) in the overlap-weighted ATO GEE. Attrition sensitivities were 10.9 points (95% CI, 3.1 to 18.7) when death was carried forward as non-examinable, 10.7 points (95% CI, 2.8 to 18.6) among patients with at least 24 h of analyzable time, and 9.8 points (95% CI, 1.7 to 17.9) for an alive-and-examinable composite (Supplementary Table S7). After adding ordered-target stratum, the difference remained 7.8 points (95% CI, 1.4 to 14.2). Among 94 patients whose landmark ordered target fell within the examinable band of RASS −2 to 0 (34 early users and 60 comparators), the adjusted difference was 9.6 points (95% CI, 2.0 to 17.2). Thus, 34/41 (82.9%) early users and 60/79 (75.9%) comparators had an ordered target within the same band used for the primary endpoint, showing substantial empirical overlap between the two endpoint layers. The primary and diagnostic estimates are summarized in Table 1 and Figure 3A. These analyses reduce the likelihood that the primary contrast was generated solely by easier prescribed targets, but they do not remove indication-related treatment selection.

Ordered-target attainment, the key secondary care-process endpoint, was 72% (IQR, 59–84%) versus 64% (IQR, 48–78%), corresponding to a median difference of 8 percentage points (95% CI, 2 to 14). The target-conditional GEE yielded a marginal difference of 7.1 points (95% CI, 1.2 to 13.0), and the shared examinable-target-band restriction yielded 74% versus 66% (difference, 8.2 points; 95% CI, 1.0 to 15.4). The observed interval-specific values also narrowed from 73% versus 62% during 24–48 h to 70% versus 65% during 48–72 h. For the primary absolute-state endpoint, the adjusted separation similarly declined from 15.2 points (95% CI, 6.0 to 24.4) at 24–48 h to 6.3 points (95% CI, −2.4 to 15.0) at 48–72 h. The corresponding 0–24 h target-attainment values (68% vs. 56%) were retained as descriptive exposure-window information only. The modeled 6-h trajectories descriptively show the greatest separation during 24–48 h, but the formal exposure-by-window interaction was imprecise (difference-in-differences, 8.9 points; 95% CI, −3.6 to 21.4; *p* = 0.164; Figure 2).

Early users had less post-landmark deep-sedation time (10% [IQR, 3–22%] vs. 21% [8–34%]; median difference, −11 points; 95% CI, −18 to −4) and more time that was both examinable and free of active propofol or midazolam (52% [34–71%] vs. 34% [18–54%]; median difference, 17 points; 95% CI, 7 to 28). During 24–72 h, propofol dose and duration differences were −5.8 mg/kg (95% CI, −13.9 to 1.0) and −6 h (95% CI, −16 to 2); corresponding midazolam and continuous-opioid dose and duration contrasts were also imprecise. These co-sedative-sparing measures are placed alongside, rather than downstream of, ordered-target attainment because they describe how the examinable state was achieved. The early-versus-never restriction was applied to examinability, ordered-target attainment, deep sedation, agitation, propofol/midazolam-free examinability, and propofol, midazolam, and continuous-opioid exposure. Directions were unchanged: adjusted examinability differed by 11.4 points (95% CI, 3.5–19.3), ordered-target attainment by 9 points (95% CI, 2–16), deep-sedation time by −12 points (95% CI, −19 to −5), and propofol/midazolam-free examinability by 18 points (95% CI, 7–29); exposure risk differences for propofol, midazolam, and continuous opioids were −18.1 points (95% CI, −35.5 to +0.6), −14.3 points (95% CI, −29.2 to +3.0), and −12.7 points (95% CI, −30.1 to +6.2), respectively (Supplementary Table S4). Alternative exposure-cutoff and carry-forward analyses also showed no direction reversal. The related estimates are shown in Figure 3, with detailed sedative-exposure results in Supplementary Figure S4. These co-sedative descriptors remain part of the observed strategy and are not independent validation of the primary endpoint or evidence of a direct pharmacologic effect.

### Hemodynamic and nimodipine-delivery safety

**Figure 4:**
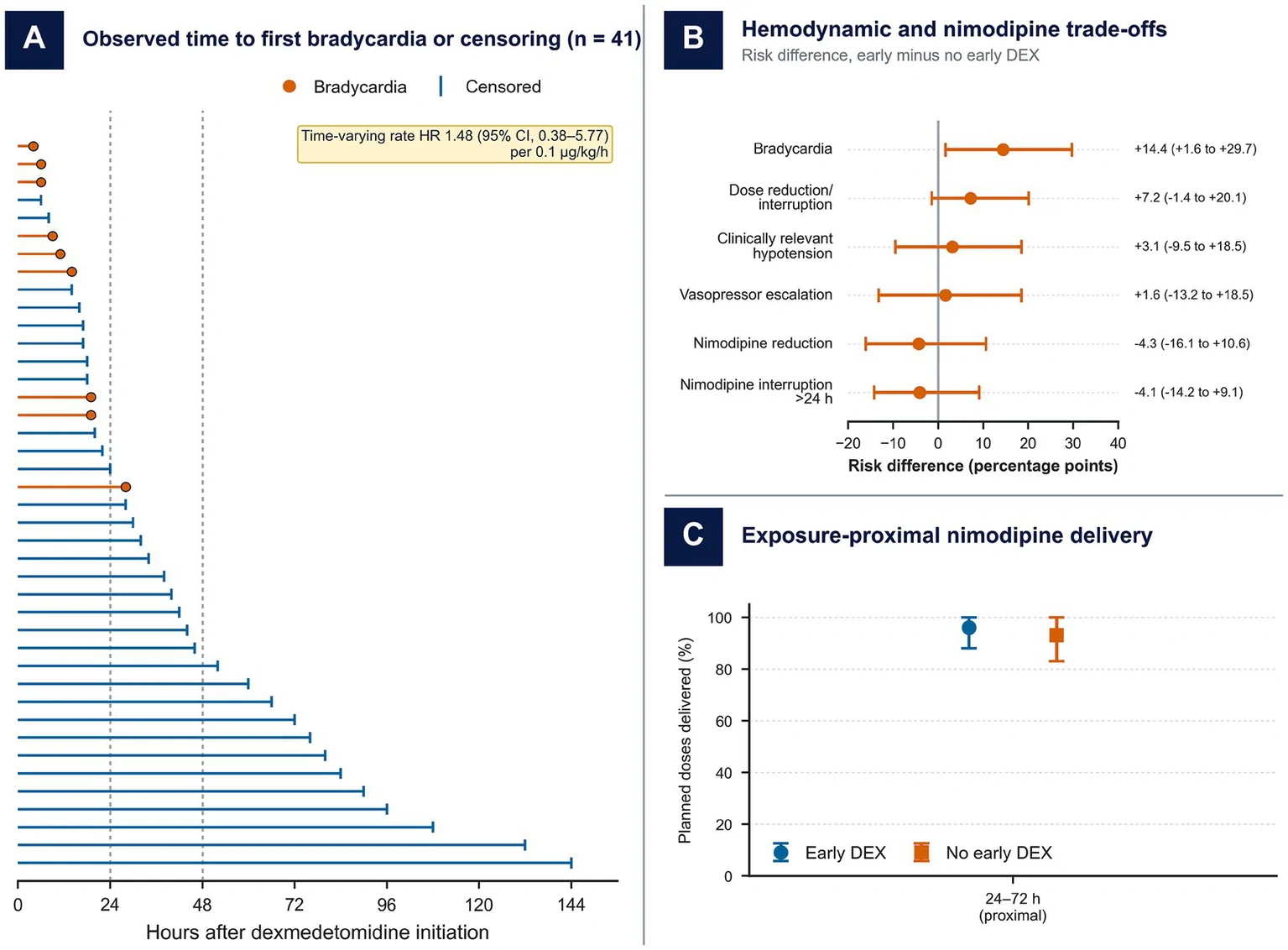
Bradycardia timing and hemodynamic/nimodipine trade-offs **(A)** Individual event-time plot among early users, showing time to first bradycardia or dexmedetomidine discontinuation/censoring without post-baseline rate stratification; the inset reports the exploratory time-varying rate model. **(B)** Risk differences, calculated as early minus no early dexmedetomidine, are shown with 95% confidence intervals for bradycardia, dose reduction or temporary interruption for bradycardia, clinically relevant hypotension, vasopressor escalation, nimodipine reduction for hypotension, and nimodipine interruption longer than 24 h. **(C)** Exposure-proximal 24–72 h nimodipine delivery on a complete 0–100% scale; points show medians and error bars show interquartile ranges. In panel A, circles denote bradycardia events, terminal ticks denote censoring, and vertical dashed lines mark 24 and 48 h after dexmedetomidine initiation.

Bradycardia occurred in 9/41 (22.0%) early users and 6/79 (7.6%) comparators (RD, 14.4 percentage points; 95% CI, 1.6 to 29.7; risk ratio, 2.89; 95% CI, 1.10 to 7.56). Four early users and two comparators required dose reduction or temporary interruption. Atropine, rescue chronotropic therapy, temporary pacing, and cardiac arrest were not recorded. Among early users, the median time from infusion initiation to first bradycardia was 11 h (IQR, 6–19), and the time-varying rate model yielded a hazard ratio of 1.48 per 0.1 micrograms/kg/h (95% CI, 0.38 to 5.77), providing no precise evidence of a dose-rate association. The individual event-time distribution and group-level safety contrasts are shown in Table 3 and Figure 4. The time-varying rate estimate was exploratory and imprecise.

**Table 3:** Bradycardia timing and hemodynamic/nimodipine-delivery outcomes.

Outcome or event	Early dexmedetomidine (*n* = 41)	No early dexmedetomidine (*n* = 79)	Effect estimate (95% CI)
Bradycardia	9 (22.0)	6 (7.6)	RD + 14.4 points (+1.6 to +29.7); RR 2.89 (1.10 to 7.56)
Lowest heart rate among bradycardia episodes, beats/min	47 (44–49)	48 (45–49)	Median difference −1 beat/min (95% CI, −4 to +2)
Dose reduction or temporary interruption for bradycardia	4 (9.8)	2 (2.5)	RD + 7.2 points (−1.4 to +20.1)
Atropine, pacing, or cardiac arrest	0 (0)	0 (0)	No events in either group
Time to first bradycardia after DEX initiation, h	11 (6–19)	Not applicable	--
Bradycardia and time-varying DEX infusion rate among early users	9 events / 41 patients	Not applicable	HR 1.48 per 0.1 micrograms/kg/h (95% CI, 0.38 to 5.77)
Clinically relevant hypotension	7 (17.1)	11 (13.9)	RD + 3.1 points (−9.5 to +18.5)
Vasopressor escalation	10 (24.4)	18 (22.8)	RD + 1.6 points (−13.2 to +18.5)
Norepinephrine-equivalent exposure, 24–72 h, micrograms/kg	0.8 (0.0–3.1)	1.0 (0.0–3.8)	Median difference −0.2 micrograms/kg (95% CI, −1.1 to +0.8)
Nimodipine reduced for hypotension	5 (12.2)	13 (16.5)	RD −4.3 percentage points (−16.1 to +10.6)
Nimodipine interruption >24 h	3 (7.3)	9 (11.4)	RD −4.1 points (−14.2 to +9.1)
Nimodipine doses delivered during 24–72 h (proximal)	96 (88–100)%	93 (83–100)%	Median difference +3 points (95% CI, −1 to +8)

Continuous variables are median (interquartile range), and categorical variables are *n* (%). Clinically relevant hypotension and nimodipine reduction are separate endpoints. Norepinephrine-equivalent exposure and nimodipine delivery are restricted to 24–72 h. The time-varying infusion-rate model reports a hazard ratio per 0.1 micrograms/kg/h and does not define a clinical threshold. DEX, dexmedetomidine; HR, hazard ratio; RD, risk difference.

Clinically relevant hypotension occurred in 17.1% versus 13.9% (RD, 3.1 percentage points; 95% CI, −9.5 to 18.5), and vasopressor escalation in 24.4% versus 22.8% (RD, 1.6; 95% CI, −13.2 to 18.5). These intervals are compatible with modest benefit or clinically important harm and do not establish equivalence. Nimodipine reduction for hypotension occurred in 12.2% versus 16.5%, and interruption longer than 24 h in 7.3% versus 11.4%. During the exposure-proximal 24–72 h window, the median proportion of planned nimodipine doses delivered was 96% versus 93% (median difference, 3 percentage points; 95% CI, −1 to 8).

### Exploratory downstream clinical outcomes

Downstream clinical outcomes are summarized in Table 4; mortality robustness is displayed in Supplementary Figure S3. Sixty-day mortality was 19.5% in the early-initiation group and 27.8% in the no-early-initiation group (RD, −8.3 percentage points; 95% CI, −22.5 to 8.6). DCI occurred in 17.1 and 19.0%, including 5 imaging-supported and 2 clinical-only diagnoses among early users and 12 imaging-supported and 3 clinical-only diagnoses among comparators, and favorable mRS 0–3 at day 60 occurred in 43.9 and 38.0%. The full day-60 mRS distribution is shown in Supplementary Figure S1. Mechanical ventilation lasted a median of 18 h longer and ICU stay 44 h longer in early users. These longer durations are compatible with residual severity and indication confounding and were not reconciled as a benefit.

**Table 4:** Exploratory downstream clinical outcomes with effect estimates.

Outcome	Early dexmedetomidine (*n* = 41)	No early dexmedetomidine (*n* = 79)	Between-group contrast
60-day all-cause mortality	8 (19.5)	22 (27.8)	RD −8.3 percentage points (95% CI, −22.5 to +8.6)
In-hospital mortality	6 (14.6)	16 (20.3)	RD −5.6 percentage points (95% CI, −18.4 to +10.0)
Delayed cerebral ischemia	7 (17.1)	15 (19.0)	RD −1.9 percentage points (95% CI, −15.1 to +14.0)
Symptomatic vasospasm	9 (22.0)	20 (25.3)	RD −3.4 percentage points (95% CI, −17.9 to +13.6)
New ischemic infarction	5 (12.2)	13 (16.5)	RD −4.3 percentage points (95% CI, −16.1 to +10.6)
Hospital-acquired infection	18 (43.9)	31 (39.2)	RD + 4.7 percentage points (95% CI, −13.2 to +22.8)
Mechanical ventilation duration, h	112 (36–240)	94 (18–170)	Median difference +18 h (95% CI, −22 to +62)
ICU length of stay, h	186 (62–280)	142 (43–266)	Median difference +44 h (95% CI, −18 to +93)
mRS 0–3 at day 60	18 (43.9)	30 (38.0)	RD + 5.9 percentage points (95% CI, −11.9 to +24.0)

Categorical outcomes are *n* (%), with Newcombe risk differences and 95% CIs; continuous outcomes are median (interquartile range), with median differences and 95% CIs. These downstream outcomes are retained for context and do not define the central sedation-state claim. DCI, delayed cerebral ischemia; DEX, dexmedetomidine; ICU, intensive care unit; mRS, modified Rankin Scale; RD, risk difference.

A descriptive stratification by mechanical ventilation status at ICU admission suggested that the longer support durations were concentrated among patients who were already ventilated at baseline: in that stratum, median ventilation duration was 144 versus 120 h and ICU stay was 216 versus 168 h, whereas among patients not ventilated at admission the corresponding medians were 0 versus 0 h and 96 versus 84 h (Supplementary Table S5). This stratification is descriptive and *post hoc* and is not an adjustment for severity.

The crude 24-h landmark mortality odds ratio was 0.63 (95% CI, 0.25–1.57) and attenuated to 0.82 (95% CI, 0.30–2.22) after overlap weighting. Excluding delayed users yielded an odds ratio of 0.85 (95% CI, 0.29–2.44). The E-value was 2.21 for the crude mortality risk ratio of 0.70 and 1.44 for the overlap-weighted estimate after approximating the common-outcome odds ratio of 0.82 as a risk ratio of 0.91; because both confidence intervals included the null, the E-value for the confidence limit closest to the null was 1.00. The exploratory falsification-style infection outcome was not lower in early users, and DCI estimates were close to null. These checks do not support a robust downstream clinical-outcome claim and are deliberately separated from the main sedation-state narrative. For the primary process association, the standardized marginal probabilities (69% vs. 57%) corresponded to an approximate risk ratio of 1.21 and a point-estimate E-value of 1.71; the approximate confidence-limit E-value was 1.35. These modest values reinforce, rather than relax, the residual-confounding caveat (Supplementary Table S3).

## Discussion

### Principal findings

In this clipped-aSAH cohort, the primary finding was greater observed post-landmark RASS-defined examinability; co-sedative measures described the associated strategy but did not independently validate it. Early users spent more post-landmark time in RASS-defined examinability, more time simultaneously examinable and free of propofol or midazolam, and had directionally lower but imprecise post-landmark propofol dose and infusion-duration estimates. Ordered-target attainment then provided a concordant care-process check: its direction persisted in the target-conditional and shared examinable-target-band analyses. Both endpoint layers showed larger descriptive contrasts during 24–48 h, but the formal interaction was imprecise and did not establish a localized window effect. Bradycardia was the clearest trade-off, while hypotension, vasopressor, and nimodipine estimates remained imprecise. Downstream outcomes did not show a stable benefit.

The contribution is therefore not a simple endpoint substitution. The primary patient-state result and the co-sedative-sparing pattern describe what state patients occupied and how that state was achieved; ordered-target attainment tests whether the unit also executed its intended sedation plan; and the safety domain identifies the cardiovascular cost. Co-sedative measures are downstream components of the same bedside strategy and therefore describe implementation, not an independent evidentiary layer.

### Endpoint hierarchy and related process measures

RASS-defined examinability is defined without reference to the order, but it is not a clinical hard endpoint and does not make treatment allocation unconfounded. Ordered-target attainment remains vulnerable to prescription preference, yet it is a legitimate quality-of-execution endpoint. Similar target distributions and monitoring density, together with a 7.8-point target-conditional primary estimate and concordant shared examinable-target-band results, make a purely easier-order explanation less likely. However, 34/41 early users and 60/79 comparators had ordered targets within RASS −2 to 0, so patient-state and target-attainment measures are related rather than empirically independent. They do not prove that dexmedetomidine caused a change in patient state, because agitation, ventilation, intracranial-pressure burden, and clinician preference may still determine both treatment and outcome.

Absolute examinability and ordered-target attainment both showed larger descriptive contrasts during 24–48 h than 48–72 h, but the exposure-by-window interaction was imprecise and the apparent attenuation may reflect chance. The temporal pattern therefore identifies an observation to test prospectively rather than a defined protocol window. Delayed dexmedetomidine use in comparators, spontaneous neurological recovery, evolving ventilation needs, target changes, and clinician-directed de-escalation remain alternative explanations. A future protocol should prospectively test whether any clinically useful temporal heterogeneity exists.

### Relation to recent literature

The recent aSAH sedation scoping review found only 11 eligible studies with a median sample size of 47 and concluded that the evidence guiding analgosedative choice is critically inadequate (6). SPRINT-SAH documented heterogeneous practice and the absence of specific protocol recommendations, while ROUTINE-SAH showed that interruption of prolonged sedation can be accompanied by frequent adverse events (7, 8). Recent dexmedetomidine studies, including administrative cohorts and a randomized dose-comparison trial, have concentrated on mortality, neurological outcomes, or biomarkers (14–17).

The present study addresses a different and more operational gap: how sedation was achieved hour by hour after clipping. The combination of time-weighted RASS-state occupancy, ordered-target care-process performance, 6-h longitudinal modeling, GABAergic-sedative displacement, proximal nimodipine delivery, and rate-localized bradycardia is not available in outcome-focused datasets. The patient-state and ordered-target endpoints are related rather than independent; the shared-band restriction makes this alignment explicit rather than providing independent validation. Their larger descriptive contrasts during the first post-landmark day were not confirmed by the formal interaction and remain hypothesis-generating. The time-resolved analysis describes when between-group differences were observed and attenuated, but the imprecise interaction does not establish temporal heterogeneity or a causal protocol window.

### Nimodipine and hemodynamic interpretation

The safety signal is clinically useful because it retains individual event timing rather than reducing the analysis to a group incidence. Bradycardia was more frequent and occurred early after initiation. The time-varying rate estimate was sparse and imprecise and did not establish a dose-rate relation or clinical threshold. The absence of atropine, pacing, or cardiac arrest is reassuring only for common severe events in this cohort; uncommon harm remains unresolved.

Nimodipine remains foundational after aSAH, and hypotension can lead clinicians to reduce or interrupt therapy (1, 2, 23, 24). The exposure-proximal 24–72 h delivery metric is the more interpretable descriptor; longer-horizon delivery extends beyond the median dexmedetomidine exposure and was not used as supporting evidence. Wide confidence intervals for hypotension, vasopressor escalation, and nimodipine modification require close heart-rate and blood-pressure surveillance, avoidance of loading doses, and readiness to reduce or pause the infusion; they do not establish universal safety.

### Downstream outcomes and residual confounding

Mortality, DCI, and functional outcome were deliberately kept outside the central claim because they reflect multiple dimensions of hemorrhage severity and care (25). The crude mortality contrast attenuated after overlap weighting and restriction, the E-values were modest, and all confidence intervals included the null. The falsification-style infection result did not show a broad healthy-user advantage, but it was imprecise and was not a strict negative control because indirect pathways through ventilation or ICU exposure remain possible.

Residual confounding may operate in opposing directions. Dexmedetomidine may be selected for patients who are ventilated, agitated, or difficult to examine, making early users appear sicker; clinicians may avoid it in patients with bradycardia, hypotension, or poor perfusion reserve, making early users appear safer. The strategy-associated process phenotype is therefore more defensible than a drug-specific or downstream-outcome claim, but it remains observational.

The apparent ventilation/ICU paradox is consistent with this indication pathway rather than contradictory to the light-sedation result. Before exposure classification, early users already had more WFNS grade IV-V disease (63.4% vs. 55.7%) and were more often mechanically ventilated at ICU admission (75.6% vs. 69.6%). These severity markers can independently increase both the probability of selecting dexmedetomidine for an intubated patient who still requires examination and the subsequent duration of ventilatory and ICU support. In the descriptive admission-ventilation stratification, the excess support time was concentrated among patients ventilated at baseline, with little separation in ventilation duration among those not ventilated at admission (Supplementary Table S5). The coherent interpretation is therefore severity - > DEX selection and severity - > longer support time; the data do not show that improved examinability shortened recovery, nor do they establish that dexmedetomidine prolonged support. For the primary process contrast, a severity pathway (greater severity - > DEX selection and lower examinability) would bias the association toward the null, whereas preferential DEX selection for patients whom clinicians wished to keep examinable could bias it away from the null; the net direction cannot be identified from these data.

### Strengths and limitations

Strengths include a time-stamped landmark construction, strict separation of exposure and outcome windows, an operational patient-state primary endpoint defined without reference to the ordered target, retention of ordered-target attainment as a key process endpoint, hour-level RASS reconstruction, 6-h GEE trajectories, target and monitoring diagnostics, shared examinable-target-band and early-versus-never restrictions, sedative dose and duration data, proximal nimodipine delivery, and time-resolved characterization of bradycardia. Joint reporting of actual state, protocol execution, and sedative displacement is more informative than any single process metric, but these measures are related.

The limitations remain substantial. This was a retrospective, single-center study limited to clipped patients, with only 41 early users. The RASS −2 to 0 primary endpoint, target-conditional models, shared examinable-target-band restriction, and propofol/midazolam-free examinable-time measure were specified *post hoc* and should be prospectively validated. RASS −2 to 0 is an operational examinability state, not a validated patient-centered or clinical hard endpoint. RASS was recorded by unblinded bedside nurses who also participated in sedative titration; RASS −2 may reflect different arousability across agents, and a brief RASS response does not guarantee an equivalent neurological examination. Although continuous cardiac monitoring was routine in both groups, dexmedetomidine exposure may have prompted closer review of heart-rate traces and therefore differential detection of short bradycardia episodes. Clinical DCI detection also depends on neurological examinability, so exposure could influence DCI ascertainment; separating imaging-supported from clinical-only diagnoses describes this concern but does not remove it. Target adjustment is diagnostic rather than causal because the ordered target may itself be influenced by the treatment strategy. The shared examinable-target-band restriction aligned the intended depth category but did not make exact ordered targets identical. Confounding by indication cannot be eliminated; intracranial-pressure burden, agitation immediately before treatment, delirium, exact examination quality, and clinician rationale may be incompletely recorded. Time weighting depends on the carry-forward rule, conditioning on remaining alive and in the ICU may bias estimates in either direction because death removes potentially non-examinable time whereas earlier ICU discharge removes potentially examinable time, the admission-ventilation stratification is descriptive and *post hoc*, the process-endpoint E-value is a secondary ratio-scale approximation, the time-varying rate analysis is sparse and imprecise, and uncommon hemodynamic harm cannot be excluded.

A prospective next step should be multicenter and protocolized, include clipped and coiled patients, prespecify both the operational examinability endpoint and ordered-target process endpoint, document the indication and physiology at every sedative change, and prospectively test whether any clinically useful temporal heterogeneity exists after the landmark. The protocol should define rescue sedation, a prospectively selected rate ceiling, bradycardia-management rules, nimodipine-preservation criteria, blinded DCI adjudication, and patient-centered outcomes. The testable hypothesis is that an early dexmedetomidine-based strategy increases actual examinable time and ordered-target attainment while reducing deep or GABAergic sedation without an unacceptable cardiovascular trade-off.

## Conclusion

After microsurgical clipping for aSAH, early dexmedetomidine was associated with more post-landmark RASS-defined examinability, less deep sedation, directionally lower but imprecise post-landmark co-sedative measures, and greater ordered-target attainment. Related process measures showed the same direction, while the apparently larger 24–48 h contrast was not confirmed by the formal interaction test; bradycardia was the principal observed trade-off. The temporal pattern is hypothesis-generating and does not define a validated sedation-strategy window, but it does not eliminate confounding by indication or establish a drug-specific causal effect, faster recovery, safety equivalence, neuroprotection, or downstream clinical-outcome benefit.

## Data Availability

The original contributions presented in the study are included in the article/Supplementary material, further inquiries can be directed to the corresponding author.

## Glossary

aSAH: Aneurysmal subarachnoid hemorrhage
APACHE II: Acute Physiology and Chronic Health Evaluation II
ATO: Average treatment effect in the overlap population
CI: Confidence interval
CT: Computed tomography
CTA: Computed tomographic angiography
DCI: Delayed cerebral ischemia
DEX: Dexmedetomidine
DSA: Digital subtraction angiography
ESS: Effective sample size
GCS: Glasgow Coma Scale
GEE: Generalized estimating equation
ICU: Intensive care unit
IQR: Interquartile range
mRS: Modified Rankin Scale
OR: Odds ratio
RASS: Richmond Agitation-Sedation Scale
RD: Risk difference
RR: Risk ratio
SAH: Subarachnoid hemorrhage
SMD: Standardized mean difference
TCD: Transcranial Doppler
WFNS: World Federation of Neurosurgical Societies
