# Supplementary material for: Early dexmedetomidine and postoperative RASS-defined examinability after microsurgical clipping for aneurysmal subarachnoid hemorrhage: a time-resolved 24-h landmark cohort study
Source: Front Neurol. 2026 Sep 4;17:1908727. doi: 10.3389/fneur.2026.1908727 (PMC13585643; doi:10.3389/fneur.2026.1908727)
Supplement: Supplementary file 1 [file Supplementary_file_1.DOCX]

**Supplementary methods and analyses**

# Supplementary Methods

## Exposure, endpoint hierarchy, and covariate handling

Early dexmedetomidine was classified during 0-24 h after clipping; primary and key secondary process outcomes were assessed during 24-72 h. The operational primary endpoint was time-weighted RASS-defined examinability (RASS -2 to 0), defined without reference to the ordered target. Ordered-target attainment was the key secondary care-process endpoint. Post-exposure co-sedative exposure and nimodipine modification were treated as components or consequences of the observed strategy, not as baseline confounders for mortality.

## Hour-level RASS reconstruction

Each RASS observation was carried forward from its charted time until the next assessment, capped at 6 h. Longer gaps, time outside ICU observation, and intervals without a classifiable RASS value were excluded from the denominator. Patient-level state occupancy and ordered-target attainment were reconstructed for consecutive 6-h bins from 24 to 72 h. Generalized estimating equations used patient clustering, an autoregressive working correlation, robust standard errors, early dexmedetomidine, time bin, an exposure-by-time interaction, and the baseline/contextual covariates specified in the main manuscript.

## Target-conditional and shared-target analyses

The target-conditional model explicitly added the ordered-target stratum active at the 24-h landmark. The shared examinable-target-band restriction included patients whose landmark ordered target was within RASS -2 to 0. These analyses were diagnostic checks for easier prescription targets; they were not treated as causal adjustment because target selection may be part of the treatment strategy.

## Overlap weighting and mortality robustness

The propensity-score model and overlap-weighting estimand are specified in the main manuscript. Extended balance, positivity, effective-sample-size, influence, DCI, E-value, and falsification-style outcome checks are reported below; mortality timing-window and complete-case checks are displayed in Supplementary Figure S3. Weighted mortality estimates remain exploratory measured-confounding checks.

## DCI review

DCI was reviewed using the consensus definition in the main manuscript, with attention to timing, sustained neurological deterioration or imaging-defined ischemia/infarction, and exclusion of rebleeding, hydrocephalus, seizure, infection, metabolic disturbance, procedure-related infarction, and sedative effect.

## Process-endpoint E-value

Because the primary process estimand is an absolute marginal probability difference, the exploratory E-value was calculated on an approximate standardized marginal risk-ratio scale. This analysis is secondary, ratio-scale dependent, and does not replace the primary difference-scale estimate. The resulting point estimate and confidence-limit approximation are exploratory and should be interpreted cautiously.

## Admission-ventilation stratification

Mechanical ventilation duration and ICU length of stay were descriptively stratified by mechanical ventilation status at ICU admission to examine whether the apparent longer-support paradox was concentrated in patients already requiring ventilatory support. This post hoc analysis is descriptive and is not an adjustment for severity.

# Supplementary Tables

**Supplementary Table S1. Operational definitions and coding rules.**

| Item | Definition used for analysis |
| --- | --- |
| Time zero | Postoperative ICU admission after aneurysm clipping. |
| Exposure window | 0-24 h after clipping. |
| Outcome window | 24-72 h after clipping for the primary and key secondary endpoints. |
| Early dexmedetomidine | Infusion started within 24 h and continued for at least 2 h. |
| Comparator | No initiation in the first 24 h; includes never users and delayed users. |
| RASS-defined examinability | RASS -2 to 0, defined without reference to the contemporaneous ordered target. |
| Ordered-target attainment | Time-weighted observed time within the contemporaneous physician/nursing ordered RASS target. |
| Carry-forward rule | Each RASS entry carried forward to the next assessment, capped at 6 h. |
| Analyzable time | Observed ICU time covered by a classifiable RASS value under the carry-forward rule. |
| Deep sedation / agitation | RASS <= -4 / RASS >= +1. |
| Bradycardia | Heart rate <50 beats/min for at least 5 consecutive minutes or prompting dose reduction, interruption, or treatment; ascertained from continuous bedside monitoring and charted vital signs. |
| Clinically relevant hypotension | SBP <90 mmHg or MAP <65 mmHg for at least 15 min and prompting fluid or vasopressor escalation; nimodipine reduction alone did not qualify. |
| Vasopressor escalation | Documented increase in vasopressor support. |
| Nimodipine modification and planned-dose delivery | Dose reduction or interruption attributed to hypotension was analyzed separately. For 24-72 h delivery, the denominator was doses scheduled by the active order while alive and hospitalized; doses after ICU transfer and tube-to-oral transition remained eligible, doses after death or hospital discharge did not, and 30-mg every-2-h dosing was converted to 60-mg every-4-h-equivalent opportunities. |
| DCI | Consensus neurological or imaging deterioration after exclusion of common mimics. |
| Favorable day-60 status | mRS 0-3. |

**Supplementary Table S2. Variable-level completeness and follow-up ascertainment.**

| Domain | Available | Handling |
| --- | --- | --- |
| Landmark exposure status | 120/120 (100%) | Complete |
| Primary RASS outcome | 120/120 (100%) | Completeness represented by analyzable-time coverage |
| Ordered RASS target at landmark | 120/120 (100%) | Complete |
| Analyzable-time coverage, median | 94% vs 92% | Uncovered time excluded from denominator |
| Propensity-score covariates | 120/120 (100%) | No missing covariates |
| Day-60 vital status | 120/120 (100%) | Complete |
| Day-60 mRS | 120/120 (100%) | Complete |
| RASS observations during 0-24 h and aligned index state | 120/120 (100%) | 0-24 h proportion descriptive; pre-initiation state based on risk-set-aligned observations |
| Co-sedative infusion records, 24-72 h | 120/120 (100%) | Complete medication-administration records |
| Hemodynamic safety outcomes | 120/120 (100%) | Continuous monitor and chart review complete |
| Nimodipine planned-dose delivery, 24-72 h | 120/120 (100%) | Active orders and administration records complete; terminal-event denominator rule applied |
| DCI diagnostic source among DCI cases | 22/22 (100%) | Imaging-supported versus clinical-only source classified |
| Prealbumin (ancillary descriptor; not in propensity score) | 113/120 (94.2%) | Available-case summary; no imputation; complete-case sensitivity n = 113 |

*Prealbumin was unavailable in 7 patients; it was not included in the propensity score, and the complete-case sensitivity included 113 patients. All primary exposure, RASS, medication, safety, and day-60 variables were complete at the patient level; within-patient RASS coverage is reported separately.*

**Supplementary Table S3. Propensity-score, weighting, and nonduplicated robustness diagnostics. Panel A. Propensity-score, overlap-weighting, and influence diagnostics.**

| Check | Result |
| --- | --- |
| Observed score range | 0.20-0.76 early; 0.08-0.73 no early. |
| Largest weighted SMD among propensity-score covariates / all displayed descriptors | <0.001 / 0.06 |
| Weight construction | No interactions, stabilization, trimming, truncation, or common-support exclusions |
| Overlap-weight distribution | Median 0.47 (IQR 0.33-0.61); range 0.12-0.86. |
| Effective sample size | 91.4. |
| Leave-one-death-out | Weighted ORs 0.72-0.96; all CIs crossed 1.00. |

*Propensity-score diagnostics are summarized with standardized mean differences, score overlap, weight distribution, and effective sample size.*

Panel B. DCI, E-value, and falsification-style analyses.

| Analysis | Comparison | Effect estimate |
| --- | --- | --- |
| DCI crude landmark | Initiation within 24 h | DCI OR 0.88 (95% CI, 0.33-2.38); P = 0.804 |
| DCI overlap weighted | 24-h landmark with overlap weights | DCI OR 0.91 (95% CI, 0.31-2.61); P = 0.859 |
| Mortality E-values | Crude RR / weighted OR | 2.21 / 1.44; confidence-limit E-values 1.00 |
| Exploratory falsification-style infection outcome | Hospital-acquired infection; not a strict negative control | RD +4.7 percentage points (95% CI, -13.2 to +22.8) |
| Primary process E-value | Approximate standardized marginal RR 1.21 | Point E-value 1.71; approximate confidence-limit E-value 1.35 |

*DCI odds ratios and the infection risk difference were calculated from reported counts. Hospital-acquired infection was a falsification-style outcome, not a strict negative control, because indirect pathways through ventilation duration or ICU exposure remain possible. The overlap-weighted mortality E-value uses an approximate risk ratio of 0.91 rather than the common-outcome odds ratio; mortality and process-endpoint E-values are exploratory sensitivity descriptors; the latter is based on an approximate ratio-scale conversion and should be interpreted cautiously.*

**Supplementary Table S4. Early dexmedetomidine versus never use: process and co-sedative restriction analyses.**

| Endpoint | Early DEX (n = 41) | Never DEX (n = 71) | Effect estimate (95% CI) |
| --- | --- | --- | --- |
| Adjusted examinability, 24-72 h | 69% | 57% | Average marginal difference +11.4 points (95% CI, +3.5 to +19.3) |
| Ordered-target attainment, 24-72 h | 72 (59-84)% | 63 (47-77)% | Median difference +9 points (95% CI, +2 to +16) |
| Deep-sedation time, 24-72 h | 10 (3-22)% | 22 (8-35)% | Median difference -12 points (95% CI, -19 to -5) |
| Agitation time, 24-72 h | 6 (0-12)% | 10 (2-20)% | Median difference -4 points (95% CI, -9 to +1) |
| Examinable time without propofol/midazolam | 52 (34-71)% | 33 (17-53)% | Median difference +18 points (95% CI, +7 to +29) |
| Propofol exposure, 24-72 h | 18 (43.9) | 44 (62.0) | RD -18.1 points (95% CI, -35.5 to +0.6) |
| Propofol cumulative dose; duration, 24-72 h | 8.6 (0-24.0) mg/kg; 10 (0-30) h | 16.3 (0-37.0) mg/kg; 20 (0-48) h | Dose difference -6.5 mg/kg (95% CI, -14.9 to +0.8); duration difference -8 h (95% CI, -18 to +1) |
| Midazolam exposure; cumulative dose; duration, 24-72 h | 8 (19.5); 0.0 (0-0.8) mg/kg; 0 (0-3) h | 24 (33.8); 0.0 (0-2.2) mg/kg; 0 (0-8) h | RD -14.3 points (95% CI, -29.2 to +3.0); dose -0.2 mg/kg (-0.8 to +0.1); duration -2 h (-8 to +1) |
| Continuous-opioid exposure; cumulative dose; duration, 24-72 h | 15 (36.6); 0.12 (0.00-0.28) morphine eq. mg/kg; 12 (0-32) h | 35 (49.3); 0.16 (0.00-0.38) morphine eq. mg/kg; 20 (0-42) h | RD -12.7 points (95% CI, -30.1 to +6.2); dose -0.04 mg/kg (-0.11 to +0.04); duration -6 h (-14 to +3) |

*Values are n (%) and median (interquartile range), as applicable.*

**Supplementary Table S5. Mechanical ventilation and ICU support duration stratified by ventilation status at ICU admission.**

| Admission stratum | Early n | No early n | Ventilation duration, h  Early | Ventilation duration, h  No early | ICU stay, h  Early | ICU stay, h  No early |
| --- | --- | --- | --- | --- | --- | --- |
| Ventilated at ICU admission | 31 | 55 | 144 (72-288) | 120 (48-216) | 216 (96-312) | 168 (72-288) |
| Not ventilated at ICU admission | 10 | 24 | 0 (0-24) | 0 (0-12) | 96 (48-192) | 84 (36-168) |

*Values are median (interquartile range). This stratification is descriptive and post hoc; it is not an adjustment for severity.*

# Supplementary Table S6. One-to-one mapping of hierarchical cohort exclusions.

| Original exclusion criterion | n | Figure 1 grouped category |
| --- | --- | --- |
| Non-aneurysmal or traumatic SAH | 14 | Nonaneurysmal or traumatic SAH |
| Age <18 years | 2 | Age <18 years |
| Ictus-to-admission time >72 h | 10 | Ictus-to-admission time >72 h |
| No clipping within 48 h or transfer | 13 | No clipping within 48 h or transfer |
| Severe pre-existing neurological disability | 2 | Severe preexisting disease/contraindication |
| Terminal organ failure | 1 | Severe preexisting disease/contraindication |
| Active malignancy | 1 | Severe preexisting disease/contraindication |
| Severe acute hepatic or renal injury | 1 | Severe preexisting disease/contraindication |
| Severe pneumonia or intracranial infection | 1 | Severe preexisting disease/contraindication |
| Recent corticosteroid/immunosuppressive exposure | 0 | Severe preexisting disease/contraindication |
| Chronic alcohol or sedative-hypnotic misuse | 0 | Severe preexisting disease/contraindication |
| Rebleeding within 7 days | 3 | Rebleeding within 7 days |
| Unavailable exposure or outcome information | 0 | No additional exclusions |

*Note. Exclusions were applied hierarchically: each screened record was assigned to the first applicable prespecified criterion and counted once. All 13 prespecified criteria are listed, and the mutually exclusive grouped counts reconcile to all 48 exclusions shown in Figure 1 (14 + 2 + 10 + 13 + 6 + 3 = 48). Zero-count criteria are retained for completeness, including unavailable exposure or outcome information (n = 0).*

# Supplementary Table S7. Second-round endpoint-validity, attrition, and ascertainment sensitivity analyses.

| Analysis | Early DEX | No early DEX | Contrast (95% CI) |
| --- | --- | --- | --- |
| RASS-defined examinability, 0-24 h | 54 (42-68)% | 51 (38-65)% | +3 points (-5 to +11) |
| Risk-set-aligned RASS -2 to 0 immediately before initiation | 20/41 (48.8%) | 43/79 (54.4%) | RD -5.6 points (-23.5 to +12.8) |
| Risk-set-aligned pre-initiation-state GEE | 68% | 58% | +10.1 points (+2.4 to +17.8) |
| Boundary sensitivity, RASS -2 to +1 | 72% | 61% | +10.6 points (+3.0 to +18.2) |
| Boundary sensitivity, RASS -3 to +1 | 84% | 75% | +8.7 points (+1.2 to +16.2) |
| Overlap-weighted ATO GEE | 68% | 57% | +10.9 points (+3.2 to +18.6) |
| Death carried forward as non-examinable | 67% | 56% | +10.9 points (+3.1 to +18.7) |
| Restricted to >=24 h analyzable time | 69% | 58% | +10.7 points (+2.8 to +18.6) |
| Alive-and-examinable composite sensitivity | 68% | 58% | +9.8 points (+1.7 to +17.9) |
| Exposure-by-window interaction | -- | -- | +8.9 points (-3.6 to +21.4); P = 0.164 |
| Documented neurological examinations, 24-72 h | 10 (8-12) | 10 (8-12) | 0 examinations (-1 to +1) |
| DCI diagnostic source | 5 imaging-supported; 2 clinical-only | 12 imaging-supported; 3 clinical-only | Descriptive |

*Notes. For the aligned index state, each early user contributed the last RASS within 2 h before DEX initiation. Each comparator received one pseudo-index hour by deterministic quantile matching to the empirical distribution of early-user initiation times and contributed the last RASS within the preceding 2 h while still untreated. The 0-24 h proportion was descriptive because it could contain post-initiation time. For the alive-and-examinable composite, death or withdrawal was coded non-examinable through 72 h and ICU discharge alive was carried forward as examinable; this was a directional sensitivity analysis.*

# Supplementary Table S8. Numbers contributing to each 6-h outcome bin and cumulative competing events.

| 6-h bin after clipping | Early DEX contributing, n | No early DEX contributing, n | Cumulative competing events |
| --- | --- | --- | --- |
| 24-30 h | 41 | 79 | None |
| 30-36 h | 41 | 78 | No early: 1 ICU discharge |
| 36-42 h | 40 | 77 | Early: 1 ICU discharge; No early: 2 ICU discharges |
| 42-48 h | 40 | 76 | No early: 2 ICU discharges, 1 death |
| 48-54 h | 39 | 75 | Early: 1 ICU discharge, 1 death; No early: 3 ICU discharges, 1 death |
| 54-60 h | 39 | 73 | No early: 5 ICU discharges, 1 death |
| 60-66 h | 38 | 71 | Early: 2 ICU discharges, 1 death; No early: 6 ICU discharges, 2 deaths |
| 66-72 h | 37 | 69 | Early: 2 ICU discharges, 1 death; No early: 7 ICU discharges, 2 deaths, 1 withdrawal decision |

# Supplementary Table S9. Post-landmark co-sedative and norepinephrine-equivalent exposure.

| Post-landmark measure, 24-72 h | Early DEX | No early DEX | Contrast (95% CI) |
| --- | --- | --- | --- |
| Propofol exposure | 18/41 (43.9%) | 47/79 (59.5%) | RD -15.6 points (-32.9 to +3.1) |
| Propofol cumulative dose; duration | 8.6 (0-24.0) mg/kg; 10 (0-30) h | 15.4 (0-35.6) mg/kg; 18 (0-46) h | -5.8 mg/kg (-13.9 to +1.0); -6 h (-16 to +2) |
| Midazolam exposure | 8/41 (19.5%) | 25/79 (31.6%) | RD -12.1 points (-26.4 to +5.0) |
| Midazolam cumulative dose; duration | 0.0 (0-0.8) mg/kg; 0 (0-3) h | 0.0 (0-2.1) mg/kg; 0 (0-8) h | -0.2 mg/kg (-0.8 to +0.1); -2 h (-8 to +1) |
| Continuous-opioid exposure | 15/41 (36.6%) | 38/79 (48.1%) | RD -11.5 points (-28.4 to +7.1) |
| Opioid cumulative dose; duration | 0.12 (0.00-0.28) morphine eq. mg/kg; 12 (0-32) h | 0.15 (0.00-0.36) morphine eq. mg/kg; 18 (0-40) h | -0.03 mg/kg (-0.10 to +0.04); -4 h (-12 to +4) |
| Norepinephrine-equivalent exposure | 0.8 (0.0-3.1) micrograms/kg | 1.0 (0.0-3.8) micrograms/kg | -0.2 micrograms/kg (-1.1 to +0.8) |

# Supplementary Figures

Supplementary Figure S1. Day-60 modified Rankin Scale distribution. Stacked bars show mRS scores 0-6 in each exposure group.

Supplementary Figure S2. Covariate balance before and after propensity-score overlap weighting. Exact balance to numerical precision was obtained for propensity-score covariates; hypertension, diabetes, aneurysm location, aneurysm diameter, white blood cell count, and prealbumin were not included in the score and are shown as untargeted descriptors.

Supplementary Figure S3. Exploratory robustness analyses for 60-day mortality. Squares indicate odds-ratio point estimates and horizontal lines indicate 95% confidence intervals for any dexmedetomidine exposure, initiation within 12 h, the primary 24-h landmark definition, initiation within 48 h, early versus never use, overlap-weighted 24-h landmark analysis, and complete-case 24-h landmark analysis. The vertical dashed line denotes the null value (OR = 1.0), and the horizontal axis is logarithmic. The primary 24-h landmark analysis is highlighted. ORs below 1 indicate lower estimated odds of mortality for the exposure definition named in that row relative to its corresponding comparator; ORs above 1 indicate higher estimated odds. All analyses are exploratory and do not eliminate residual confounding.

Supplementary Figure S4. Co-sedative exposure, dose, and duration in the early dexmedetomidine and no-early-dexmedetomidine groups. (A) Risk differences (early minus no early) with 95% confidence intervals for propofol, midazolam, and continuous-opioid exposure during 24-72 h. (B) Median differences (early minus no early) with 95% confidence intervals for 24-72 h cumulative dose and infusion duration for propofol, midazolam, and continuous opioids. Circles indicate point estimates, horizontal lines indicate 95% confidence intervals, and vertical dashed lines denote zero. Negative values indicate lower exposure, dose, or duration in the early dexmedetomidine group; positive values indicate greater exposure, dose, or duration. Dose is expressed in mg/kg and duration in hours. These descriptive contrasts characterize the observed sedation strategy and should not be interpreted as isolated pharmacologic effects.

# Completed STROBE Checklist

**STROBE checklist for cohort studies.**

| **Item** | **Recommendation** | **Reported information** | **Location** |
| --- | --- | --- | --- |
| 1 | Title and abstract | Indicate study design and provide an informative summary. | Title; Abstract |
| 2 | Background/rationale | Explain scientific background and rationale. | Introduction |
| 3 | Objectives | State specific objectives and prespecified hypotheses. | Introduction; What this study adds |
| 4 | Study design | Present key design elements early. | Methods—Study design and setting |
| 5 | Setting | Describe setting, locations, and relevant dates. | Methods—Study design and setting |
| 6 | Participants | Give eligibility, selection, and follow-up methods. | Methods—Participants; Figure 1 |
| 7 | Variables | Clearly define outcomes, exposures, predictors, and confounders. | Methods—Endpoint hierarchy; Table 2; Tables S1-S2 |
| 8 | Data sources/measurement | Describe sources and assessment methods. | Methods—Perioperative care; Sedation-state outcomes |
| 9 | Bias | Describe efforts to address bias. | Landmark design; target diagnostics; overlap weighting; restrictions |
| 10 | Study size | Explain study-size determination. | Methods—Statistical analysis |
| 11 | Quantitative variables | Explain handling of quantitative variables. | Methods—Statistical analysis |
| 12a | Statistical methods | Describe all statistical methods. | Methods—Statistical analysis |
| 12b | Subgroups/interactions | Describe subgroup and interaction methods. | Methods; Table 2; Tables S4-S5 |
| 12c | Missing data | Explain handling of missing data. | Methods; Table S2 |
| 12d | Loss to follow-up | Explain follow-up handling. | Methods—Participants; Table S2 |
| 12e | Sensitivity analyses | Describe sensitivity analyses. | Methods; Table 2; Tables S3-S4; Figures S2-S3 |
| 13 | Participants | Report numbers at each stage and reasons. | Figure 1 |
| 14 | Descriptive data | Report participant characteristics and missingness. | Table 1; Table S2 |
| 15 | Outcome data | Report numbers or summary measures. | Tables 2-4; Figure S1 |
| 16 | Main results | Give unadjusted/adjusted estimates and precision. | Results; Tables 2-4 |
| 17 | Other analyses | Report sensitivity and subgroup analyses. | Table 2; Tables S3-S5; Figures S2-S4 |
| 18 | Key results | Summarize key results with objectives. | Discussion—Principal findings |
| 19 | Limitations | Discuss limitations and direction/magnitude of bias. | Discussion—Strengths and limitations |
| 20 | Interpretation | Provide cautious overall interpretation. | Discussion; Conclusion |
| 21 | Generalisability | Discuss external validity. | Discussion—Strengths and limitations |
| 22 | Funding | State funding and funder role. | Funding statement |
